# Genome characterization of a uropathogenic *Pseudomonas aeruginosa* isolate PA_HN002 with cyclic di-GMP-dependent hyper-biofilm production

**DOI:** 10.3389/fcimb.2022.956445

**Published:** 2022-08-02

**Authors:** Siying Lin, Shuzhen Chen, Li Li, Huiluo Cao, Ting Li, Ming Hu, Lisheng Liao, Lian-Hui Zhang, Zeling Xu

**Affiliations:** ^1^ Guangdong Province Key Laboratory of Microbial Signals and Disease Control, Integrative Microbiology Research Centre, South China Agricultural University, Guangzhou, China; ^2^ Women and Children’s Health Institute, Guangdong Women and Children Hospital, Guangzhou, China; ^3^ Department of Microbiology, Li Ka Shing Faculty of Medicine, The University of Hong Kong, Hong Kong, Hong Kong SAR, China

**Keywords:** *Pseudomonas aeruginosa*, motility, biofilm, c-di-GMP, DGC, PDE

## Abstract

*Pseudomonas aeruginosa* can cause various types of infections and is one of the most ubiquitous antibiotic-resistant pathogens found in healthcare settings. It is capable of adapting to adverse conditions by transforming its motile lifestyle to a sessile biofilm lifestyle, which induces a steady state of chronic infection. However, mechanisms triggering the lifestyle transition of *P. aeruginosa* strains with clinical significance are not very clear. In this study, we reported a recently isolated uropathogenic hyper-biofilm producer PA_HN002 and characterized its genome to explore genetic factors that may promote its transition into the biofilm lifestyle. We first showed that high intracellular c-di-GMP content in PA_HN002 gave rise to its attenuated motilities and extraordinary strong biofilm. Reducing the intracellular c-di-GMP content by overexpressing phosphodiesterases (PDEs) such as BifA or W909_14950 converted the biofilm and motility phenotypes. Whole genome sequencing and comprehensive analysis of all the c-di-GMP metabolizing enzymes led to the identification of multiple mutations within PDEs. Gene expression assays further indicated that the shifted expression profile of c-di-GMP metabolizing enzymes in PA_HN002 might mainly contribute to its elevated production of intracellular c-di-GMP and enhanced biofilm formation. Moreover, mobile genetic elements which might interfere the endogenous regulatory network of c-di-GMP metabolism in PA_HN002 were analyzed. This study showed a reprogrammed expression profile of c-di-GMP metabolizing enzymes which may promote the pathoadaption of clinical *P. aeruginosa* into biofilm producers.

## Introduction

*Pseudomonas aeruginosa* is a ubiquitous opportunistic pathogen that can cause a variety of infections such as the bloodstream infection, urinary tract infection, burn wound infection and surgical site infection in human bodies (Litwin et al., 2021). Prevention and treatment of its infections are frequently hindered owing to its remarkable capacity of resisting antibiotics, and recurrence of its infections is the leading cause of morbidity and mortality in immunocompromised patients (Moradali et al., 2017; Pang et al., 2019). *P. aeruginosa* has a large genome which generally consists of ~6,000 genes with a high proportion of them encoding regulatory proteins, metabolic enzymes, outer membrane proteins involved in adhesion, motility, transportation, and signal sensing, enabling a surprising ecological versatility and adaptability to the environmental changes (Stover et al., 2000; Klockgether et al., 2011; Cao et al., 2017; Rossi et al., 2021). In addition, *P. aeruginosa* possesses a large number of genes encoding virulence factors such as phenazines, siderophores, elastase, proteases, exotoxins and cytotoxins, flagella and pili, and all these contribute significantly to its pathogenesis (Ahator and Zhang, 2019; Alonso et al., 2020).

*P. aeruginosa* is a motile microorganism and its motility is mainly driven by the polar monotrichous flagellum or the type IV pili (Toutain Christine et al., 2005; Burrows, 2012). Directed motility known as chemotaxis is beneficial for bacteria to migrate towards nutrients or evade from adverse environments such as the presence of toxins and predators. Motility represents an important virulence factor in *P. aeruginosa* to cause acute infection because it allows the pathogen to rapidly attach to and colonize surfaces of host tissues (Josenhans and Suerbaum, 2002; Kühn et al., 2021). There are three well-characterized types of motilities in *P. aeruginosa*: twitching, swarming and swimming. Twitching is a type IV pili-dependent form of multicellular motility by which *P. aeruginosa* cells move over solid surfaces (Kearns Daniel et al., 2001). Swarming occurs on semisolid surfaces (0.5-0.7% agar) which is also a multicellular phenomenon but depends on the functional flagellum as well as two biosurfactants rhamnolipids (RL) and 3-hydroxyalkanoic acids (HAA) (Ha et al., 2014a). In contrast, swimming is a unicellular movement behavior in liquid or low-viscosity conditions (up to 0.3% agar) which only requires a functional flagellum with its motor-stator complex (Ha et al., 2014b). Motilities in *P. aeruginosa* are tightly regulated. For example, the flagellum-based swimming and swarming motilities are largely dependent on the intracellular level of the global second messenger cyclic di-GMP (c-di-GMP) which further interconnects a broad spectrum of regulatory pathways such as the FleS/FleR two-component system, Gac/Rsm signaling cascade, Chp chemotaxis system, Wsp chemosensory pathway and the HptB pathway (Wolfe Alan and Visick Karen, 2008; Porter et al., 2011; Xin et al., 2019; Zhou et al., 2021).

As a member of the highly virulent and antibiotic-resistant bacterial group ESKAPE (*Enterococcus faecium*, *Staphylococcus aureus*, *Klebsiella pneumoniae*, *Acinetobacter baumannii*, *P. aeruginosa*, and *Enterobacter* spp.) (Mulani et al., 2019), *P. aeruginosa* exhibits resistance to a broad spectrum of antibiotics including β-lactams, quinolones and aminoglycosides with its intrinsic, acquired and adaptive resistance machineries (Hancock and Speert, 2000). For instance, *P. aeruginosa* intrinsically develops restricted outer membrane permeability, synthesizes a number of antibiotic-inactivating enzymes such as β-lactamases, and produces abundant efflux systems to minimize or obliterate the intruded antibiotics (Breidenstein et al., 2011). What’s worse, *P. aeruginosa* frequently elevates the refractoriness to antibiotic treatments by forming biofilms, leading to a higher rate of therapeutic failure and a state of recurrent or chronic infections (Mulcahy et al., 2014). A biofilm is described as an aggregation of microorganisms that are encased within a self-produced matrix of extracellular polymeric substances (EPSs) and adhere to each other on biotic or abiotic surfaces (Chang, 2018). The matrix of EPSs in *P. aeruginosa* biofilms is basically composed of polysaccharides, proteins, extracellular DNA (eDNA) and rhamnolipids (Mann and Wozniak, 2012). Formation of biofilm represents one of the best characterized mechanisms of adaptive resistance in *P. aeruginosa*, which protects the microbial cells inside to resist antibiotics with up to 1,000-fold greater compared to the planktonic cells and is regarded as one of the typical features during chronic infections (Mah et al., 2003; Taylor et al., 2014). Biofilm formation in *P. aeruginosa* are known to be regulated by a variety of cellular factors such as c-di-GMP, cAMP, quorum sensing (QS) systems and two-component systems (Sakuragi and Kolter, 2007; Dong et al., 2008; Almblad et al., 2019). In recent years, increased intracellular c-di-GMP level has been reported to promote biofilm formation of *P. aeruginosa* in a range of clinically important models and delay healing of wound infections caused by *P. aeruginosa* (Wei et al., 2019; Zhang et al., 2020; Li et al., 2021).

So far, extensive efforts have been made to understand the mechanisms underlying the regulation of motility and biofilm formation in *P. aeruginosa*, and c-di-GMP is found to play a critical role in the transition between the motile and sessile lifestyles, leading to the acute-to-chronic virulence switch. For instance, a high c-di-GMP level promotes sessile biofilm lifestyle while a low level of c-di-GMP is associated with the active motility (Li et al., 2017). However, genetic basis and environmental cues that trigger the shift of intracellular c-di-GMP levels and consequently induce the transition of lifestyles are still largely unknown especially in the isolates with clinical significance. In this study, we isolated a uropathogenic *P. aeruginosa* strain PA_HN002 which was found as a hyper-biofilm producer and its biofilm formation was confirmed to be dependent on the high intracellular c-di-GMP concentration. We analyzed its genome and performed a comprehensive analysis of c-di-GMP metabolizing enzymes between PA_HN002 and the reference strain PAO1, which realized that the altered expression profile of c-di-GMP metabolizing enzymes in PA_HN002 mainly induced its biofilm lifestyle in this strain. These findings highlighted the complex regulation of c-di-GMP metabolism and its importance to promote *P. aeruginosa* to adapt and persist to the clinically relevant environments.

## Materials and methods

### Strains and growth conditions

*P. aeruginosa* PA_HN002 was isolated from a mid-stream urine sample of a one-year-old pediatric patient in Guangdong Women and Children Hospital (Guangzhou, China) with the approvement from the Medical Ethical Committee of the hospital under number of 202201011. P*. aeruginosa* strains were routinely grown at 37°C in Luria-Bertani (LB) broth (Tryptone 10g/L, Yeast extract 5g/L, NaCl 10g/L). Gentamycin was supplemented in the medium when necessary: 25 μg/mL for *E. coli* DH5α and 50 μg/mL for *P. aeruginosa*.

### Genome sequencing and bioinformatic analysis

Genomic DNA of PA_HN002 was sequenced on an Illumina Hiseq platform (Sangon, China). Genome sequences were assembled, and gap filling was performed by SPAdes, GapFiller and PrInSeS-G (Massouras et al., 2010; Nadalin et al., 2012; Prjibelski et al., 2020). Gene annotation was conducted using Prokka v1.14.5 (Seemann, 2014). Protein sequences of PA_HN002 were searched against the Clusters of Orthologous Groups (COG) database with an e-value<1e-5 using blastp and results were parsed using in-house python script to summarize proteins present exclusively or shared between different *P. aeruginosa* strains. Venn diagram was generated by Venny v2.1.0 (https://bioinfogp.cnb.csic.es/tools/venny/index.html). The MLST and serotype of PA_HN002 was predicted based on the PubMLST database and the *Pseudomonas aeruginosa* serotyper (PAst) tool, respectively (Thrane Sandra et al., 2016; Jolley et al., 2018). The phylogenetic tree was built based on the alignment of 400 marker genes in PA_HN002 and 6,800 *P. aeruginosa* genomes retrieved from GenBank with fasttree using PhyloPhlAn version 3.0 and visualized using iTOL (Asnicar et al., 2020; Letunic and Bork, 2021). IME was predicted with ICEberg 2.0 (Liu et al., 2019). Insertion sequences in the PA_HN002 genome were predicted by ISFinder (Siguier et al., 2006). Prophages were predicted using the online software PHASTER (Arndt et al., 2016). Genomic islands (GIs) were predicted using IslandViewer 4 (Bertelli et al., 2017). Domain architectures of the c-di-GMP metabolizing enzymes were determined based on the SMART algorithm (Letunic et al., 2021).

### Construction of plasmids for gene expression

Genes for complementation or overexpression were amplified by PCR with the primers in **Table S1** and ligated into the downstream of the *lac* promoter of pBBR1-MCS5 between the HindIII and BamHI sites. The constructed plasmids were verified by PCR and DNA sequencing. Constructed plasmids were introduced into PA_HN002 by electroporation. Successful plasmid delivery into PA_HN002 was confirmed by PCR.

### Motility assay

Different agar plates were prepared for swimming and swarming motilities. For swimming motility, semisolid agar plates were prepared with 5 g/L Becto-peptone, 3 g/L Yeast extract and 2 g/L Becto-agar. For swarming motility, plates were prepared by 8 g/L Nutrient broth, 5 g/L Glucose and 6 g/L Becto-agar. 15 ml agar medium was poured into a 90-mm petri dish and then solidified at room temperature. Overnight bacterial culture was 1:50 diluted and grown to OD_600_ of 1.0. 2 μl cell culture was spotted into the center of the pre-dried swimming and swarming plates. The plates were incubated at 37°C for 48 hours, and images were recorded. The assays were performed in triplicates.

### Biofilm assay

Biofilm assay was performed as previously described (Cao et al., 2019). Overnight bacterial culture was 1:50 diluted and grown to OD_600_ of 1.0. After 1:50 dilution, After 1:50 dilution, 1 ml cell culture was transferred to the 24-well plate and incubated statically at 37°C for 24 h. Planktonic cells were discarded and the biofilm cells was washed carefully with MilliQ water. The plate was air dried and then stained with 2 ml 0.1% crystal violet for 15 min. After the crystal violet was removed, biofilm was washed with MilliQ water , air dried, and dissolved in 2 ml of a mixture of glacial acetic acid and 95% ethanol (1:4). Absorbance at 595 nm was measured after 10-fold dilution in a microplate reader (BioTek).

### c-di-GMP measurement by liquid chromatography-mass spectrometry

c-di-GMP was measured following a previous study with slight modifications (Chen et al., 2022). Overnight bacterial culture was 1:50 diluted and grown to OD_600_ of 1.0. 1 ml cell culture was harvested. Cell lysis was performed by adding perchloric acid (70%, v/v) with a final concentration of 0.6 M for 30 min at 4°C. Samples were centrifuged at 12,000 rpm for 10 min and the supernatants were collected. After the pH was neutralized by adding 1/5 volume of 2.5 M KHCO_3_, samples were centrifuged at 4,000 rpm for 10 min at 4°C and the supernatants were collected for LC-MS analysis. LC-MS was undertaken using a Q Exactive Focus Hybrid Quadrupole-Orbitrap mass spectrometer (Thermo Fisher Scientific) with a 1.8 μm, 100×2.1 mm high-strength silica (HSS) T3 column (Waters). 5 μl sample was injected and the m/z 691>248 transition was used for quantification.

### Reverse transcription-quantitative PCR

RT-qPCR was performed as previously described (Xu et al., 2019). Primers used for this assay were listed in **Table S1**. After the overnight culture was 1:50 diluted and grown to OD_600_ of 1.0, 1 ml cell culture was collected. Total RNA was extracted using the Eastep^®^ Super Total RNA Extraction Kit (Promega) following the manufacturer’s instructions. Reverse transcription was conducted to obtain cDNA using the TransScript^®^ One-Step gDNA Removal and cDNA Synthesis SuperMix (TransGen). qPCR was performed using the ChamQ Universal SYBR qPCR Master Mix (Vazyme). Relative gene expression was calculated based on the 2^-ΔΔCt^ method with the *recA* gene as the internal reference control (Schmittgen and Livak, 2008).

### *Galleria mellonella* Killing assay

Time-killing of *G. mellonella* was performed to evaluate the acute virulence of *P. aeruginosa* following a previous study with slight modifications (Si et al., 2017). Overnight bacterial culture was 1:50 diluted and grown to OD_600_ of 1.0. Cells were harvested and resuspended with fresh LB medium to a final concentration of 10^5^ cells per ml. Fifteen *G. mellonella* larvae were injected with 10 μl cell suspension at the secondary right proleg using a syringe and then incubated at 30°C. The survival rate of *G. mellonella* was recorded at different time points within 2 days.

## Results

### A red-pigmented clinical *P. aeruginosa* isolate PA_HN002 showed chronic infection phenotypes

**Figure 1:**
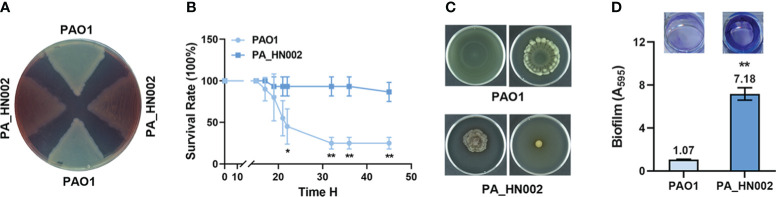
Phenotypic comparison between *P. aeruginosa* PAO1 and PA_HN002. **(A)** PA_HN002 produced red-colored pigments. **(B)** Pathogenicity of PAO1 and PA_HN002 was measured in a *G. mellonella* acute infection model. **(C)** Swimming (left) and swarming (right) motilities were measured in PAO1 and PA_HN002. Representative pictures of motilities for each strain were shown. **(D)** Biofilm formation of PAO1 and PA_HN002. *P<0.05, **P<0.01 based on Student’s *t*-test.

A *P. aeruginosa* strain PA_HN002 was recently isolated from a mid-stream urine sample of a one-year-old pediatric patient in Guangdong Women and Children Hospital (Guangzhou, China). The strain was identified as *P. aeruginosa* by matrix-assisted laser desorption ionization-time of flight mass spectrometry (MALDI-TOF-MS) and 16S rDNA sequencing. Unlike common blue/green-colored *P. aeruginosa* cultures due to the production of pyocyanin, PA_HN002 was a rarely seen *P. aeruginosa* isolate producing a red/brown-colored pigment (**Figure 1A**) which was supposed to be pyorubin (red) or pyomelanin (brown) as described previously (Rodríguez-Rojas et al., 2009; Orlandi et al., 2015). Compared to the reference strain PAO1, PA_HN002 displayed significantly attenuated *in vivo* virulence when examined using the acute infection model of *Galleria mellonella* (**Figure 1B**). Although twitching motility was comparable between PA_HN002 and PAO1 (**Figure S1**), flagellum-based swimming and swarming motilities of PA_HN002 were substantially diminished (**Figure 1C**). In contrast, extraordinary greater capacity of biofilm formation was observed in PA_HN002 with approximately 7-fold higher of biofilm biomass than that in PAO1 (**Figure 1D**). These traits indicated that PA_HN002 was associated with chronic urinary tract infection (UTI) and could serve as an important model to investigate molecular mechanisms underlying chronic adaptation of clinical *P. aeruginosa* strains.

### The high intracellular c-di-GMP level contributed to the transition from motile to sessile lifestyles in PA_HN002

**Figure 2:**
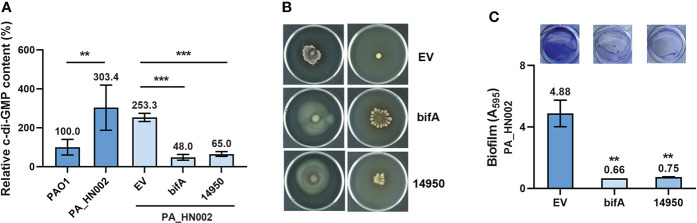
PA_HN002 displayed a c-di-GMP-dependent transition of motile-sessile lifestyles. **(A)** LC-MS quantification of the intracellular c-di-GMP content in PAO1, PA_HN002 and PA_HN002 expressing plasmid-carried PDE genes *bifA* or W909_14950 (14950). Relative content was shown in the PA_HN002 strains compared to PAO1. **(B)** Swimming (left) and swarming (right) motilities of PA_HN002 and PA_HN002 with expression of the PDE genes *bifA* and W909_14950 (14950). **(C)** Biofilm formation of PA_HN002 and PA_HN002 with expression of the PDE genes *bifA* and W909_14950 (14950). **P<0.01, ***P<0.001 based on Student’s *t*-test in **(A)** and one-way ANOVA compared to the EV (empty vector) group in **(C)**.

Motile and sessile lifestyles are the dominant characteristics of acute and chronic infections in *P. aeruginosa*, respectively, which are regulated by a variety of cellular factors and dynamics of the intracellular second messenger c-di-GMP pool was known to play a critical role in the transition of these two lifestyles (Rasamiravaka et al., 2015; Valentini and Filloux, 2016; Ciofu and Tolker-Nielsen, 2019). As speculated, LC-MS measurement showed that the intracellular c-di-GMP content in PA_HN002 was about 3-fold higher than PAO1 (**Figure 2A**). To validate whether the attenuated motilities and strong biofilm phenotypes of PA_HN002 were ascribed to its high intracellular c-di-GMP content, we introduced two plasmids expressing two well-characterized c-di-GMP hydrolysis proteins BifA and W909_14950, respectively, into PA_HN002. BifA is a hybrid protein containing both a c-di-GMP biosynthesis and hydrolysis domains from PAO1 but only exhibits the degradation activity and the other enzyme W909_14950 from *Dickeya zeae* EC1 only contains a c-di-GMP hydrolysis domain (Kuchma Sherry et al., 2007; Chen et al., 2020b). Activities of both enzymes to reduce intracellular c-di-GMP content were confirmed in two recent studies (Zhou et al., 2021; Zhou et al., 2022). As shown in **Figure 2A**, introduction of either *bifA* or W909_14950 resulted in 4~5-fold decrease of the intracellular c-di-GMP content in PA_HN002. Recovered swimming and swarming motilities and inhibited biofilm formation were observed along with the decrease of intracellular c-di-GMP content (**Figures 2B, C**). These results demonstrated that high intracellular c-di-GMP content is the key in PA_HN002 to induce the transition from its motile lifestyle to the aggregated biofilm lifestyle.

### Whole genome sequencing and general genomic characteristics of PA_HN002

**Figure 3:**
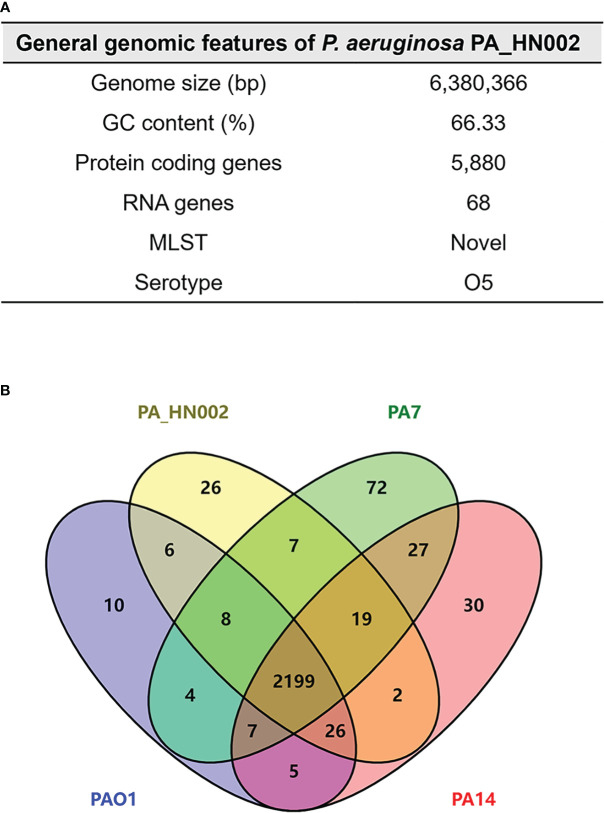
General features of the PA_HN002 genome. **(A)** General features of the PA_HN002 genome including genome size, GC content, protein coding genes, RNA genes, MLST and serotype. **(B)** Venn diagram showing the number of shared and exclusive genes among PA_HN002, PAO1, PA7 and PA14. The numbers indicated the unique genes, shared genes among two, three or four strains based on the COG gene annotations.

**Figure 4:**
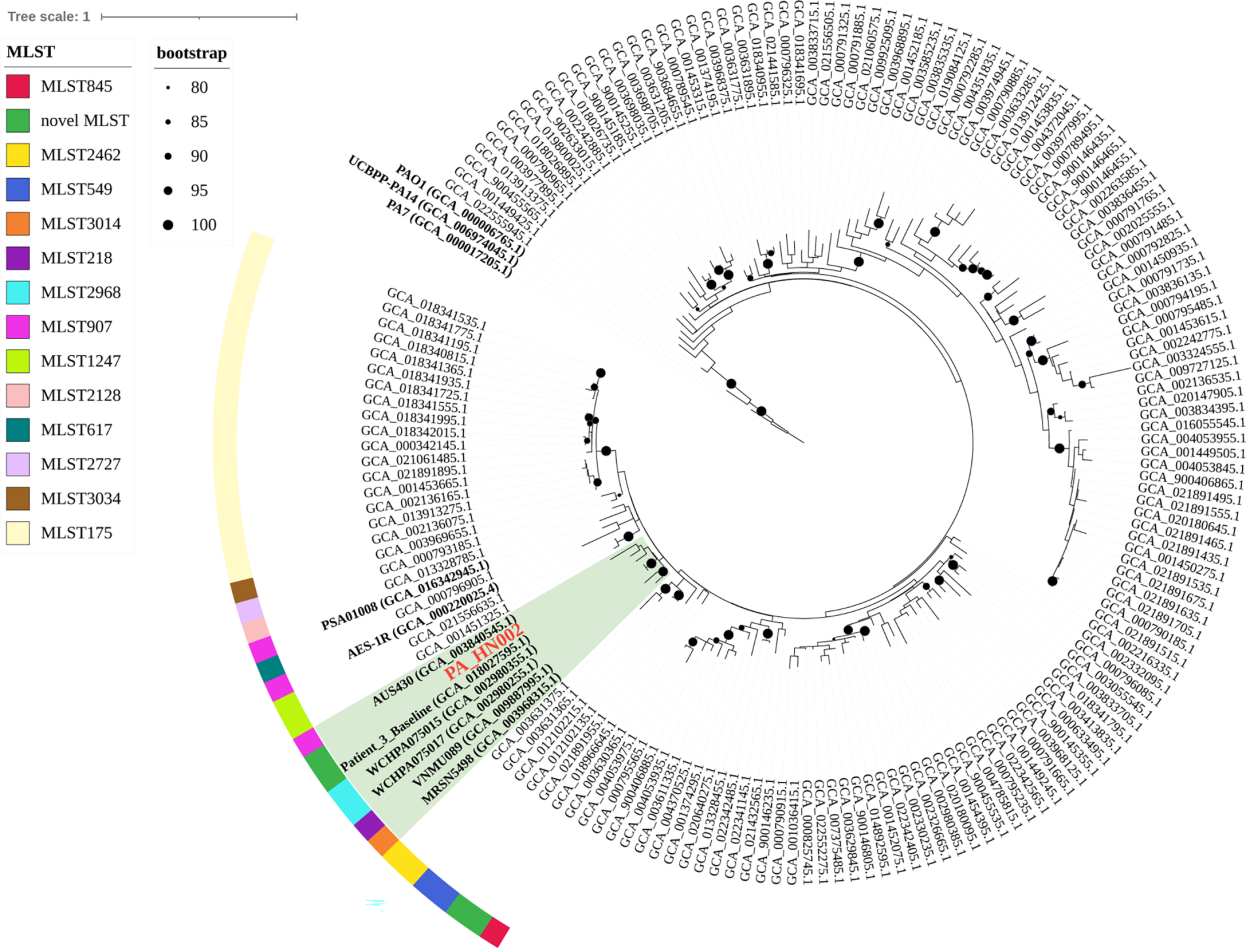
Phylogenetic position of PA_HN002. The phylogenetic tree was constructed based on 6,800 *P. aeruginosa* genomes from GenBank using PhyloPhlAn version 3.0 and the most related 168 genomes to PA_HN002 were presented. Three widely used reference genomes of PAO1, PA7 and PA14 were included and shown. Genomes located within the same clade as PA_HN002 were shaded in green. MLST types were shown in different colors for the genomes that were close to PA_HN002. Black dots on the nodes were bootstrap values and those greater than 80 were shown.

To understand the molecular basis of high c-di-GMP production in PA_HN002, genomic DNA of PA_HN002 was extracted and sequenced. Generally, a 6,380,366-bp genome with 66.33% GC content was assembled (**Figure 3A**), which shared a similar GC content but was ~0.12-Mb larger than the PAO1 genome (PAO1: 66.6% GC content and 6,26-Mb genome size). The PA_HN002 genome was predicted to contain 5,880 protein coding genes with 4,947 of them were annotated in COG (Clusters of Orthologous Groups of proteins). We compared COG annotations of PA_HN002 with those of PAO1 and another two widely used *P. aeruginosa* reference strains PA7, a non-respiratory human isolate from Argentina (Roy et al., 2010), and PA14, a highly virulent clinical isolate (He et al., 2004). A total of 26 COGs were exclusively present in PA_HN002 (**Figure 3B**). PubMLST analysis showed that PA_HN002 did not match any existing multiple locus sequence type (MLST), but with one variant allele from ST804 and ST907 which contained isolates from sputum and urinary tract infection, respectively, in Australia according to the allelic profiles. ST804 was noted as a predominant clonal complex in 1990s with an obvious phenotype of multidrug resistance (De Oliveira Santos et al., 2019), while the other one was lacking of documentation. PAst analysis showed that PA_HN002 and PAO1 belonged to the same serotype O5 which is prevalent in burn wound infections (Nasrin et al., 2022). To understand the phylogenetic relationship of PA_HN002 with other *P. aeruginosa* strains, we conducted a comprehensive phylogenetic analysis of PA_HN002 with 6,800 assembled genomes from GenBank (data not shown) and 172 genomes (**Table S2**) which included the most related 168 genomes to PA_HN002 and three reference genomes of PAO1, PA7 and PA14 were presented in **Figure 4**. It showed that PA_HN002 was closely related to six clinically isolated strains from Canada (AUS430: ST907), USA (Patient_3_Baseline: same ST as PA_HN002; VNMU089: ST218; MRSN5498: ST3014) and China (WCHPA075015: ST2968; WCHPA075017: ST2968).

### Mutations identified in the c-di-GMP metabolizing enzymes in PA_HN002 did not lead to its enhanced biofilm formation

**Figure 5:**
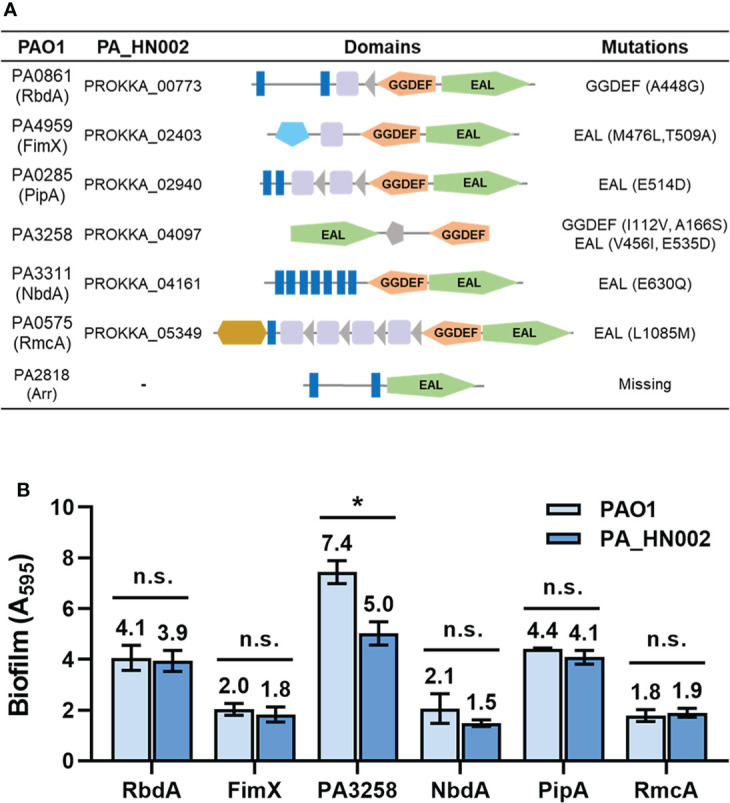
Mutations in the c-di-GMP metabolizing enzymes from PA_HN002 relative to those from PAO1. **(A)** A **s**ummary of mutations within c-di-GMP metabolizing enzymes PA0861 (RbdA), PA4959 (FimX), PA0285 (PipA), PA3258, PA3311 (NbdA), PA0575 (RmcA) and PA2818 (Arr) in PA_HN002 relative to the sequences in PAO1. **(B)** Biofilm formation of the PA_HN002 strain with overexpression of the PA0861 (RbdA), PA4959 (FimX), PA0285 (PipA), PA3258, PA3311 (NbdA), PA0575 (RmcA) encoding genes from PAO1 and PA_HN002, respectively. n.s., not significant, *P<0.05 based on Student’s *t*-test.

Intracellular c-di-GMP level is modulated by the rate of two reactions. One is its synthesis by condensation of two guanosine triphosphate (GTP) molecules and the other is hydrolysis of it into 5′-phosphoguanylyl-(3′-5′)-guanosine (pGpG) or guanosine monophosphate (GMP). Synthesis of c-di-GMP is achieved by diguanylate cyclases (DGCs) which contain a characteristic conserved GGDEF domain that forms the active site of the enzymes while hydrolysis requires the PDEs which contain an EAL or an HD-GYP domain in the active site (Ryjenkov Dmitri et al., 2005; Schmidt Andrew et al., 2005; Tchigvintsev et al., 2010; Stelitano et al., 2013). In total 41 genes encoding the c-di-GMP metabolizing enzymes in PA_HN002 were predicted from its sequenced genome based on Pfam blast using the above-mentioned three domains and the reported c-di-GMP metabolizing enzymes in PAO1 (**Table S3**). A systematic comparative analysis of these genes between PA_HN002 and PAO1 was conducted, which revealed 10 amino acid substitutions in the GGDEF or EAL domains within six GGDEF and EAL hybrid enzymes (**Figure 5A**). Specifically, three mutations were found in the GGDEF domains (A448G in PA0861 (RbdA), I112V and A166S in PA3258) and seven mutations were found in the EAL domains (M476L and T509A in PA4959 (FimX), E514D in PA0285 (PipA), V456I and E535D in PA3258, E630Q in PA3311 (NbdA), L1085M in PA0575 (RmcA)). Interestingly, all these enzymes were reported to exhibit PDE activities and some of them have been demonstrated to be associated with the biofilm formation in *P. aeruginosa* (Kazmierczak et al., 2006; Monds et al., 2007; An et al., 2010; Li et al., 2013; Katharios-Lanwermeyer et al., 2021; Cai et al., 2022). For example, RbdA and NbdA have been reported as being important for biofilm dispersion and RmcA mutant displayed a great ability to initiate a robust biofilm (Li et al., 2013; Katharios-Lanwermeyer et al., 2021). PA3258 is a putative orthologue of RapA in *P. fluorescens* which was shown to be involved in c-di-GMP degradation and inhibition of biofilm formation in *P. aeruginosa* (Monds et al., 2007; Haddad et al., 2009). FimX is also a c-di-GMP binding protein promoting type IV pili assembly and twitching motility, which is involved in the process of biofilm development (O’toole and Kolter, 1998; Jain et al., 2017). PA0285 was recently renamed as PipA (Phage inducing phosphodiesterase A) owing to its function to positively regulate phage production in PAO1 in addition to inhibit biofilm formation (Cai et al., 2022).

These mutations especially those in the EAL domains were speculated to have high possibilities of reducing the PDE activities and consequently enhancing biofilm formation. To verify whether these identified mutations contributed to the enhanced biofilm formation in PA_HN002, we cloned the six pairs of genes from PAO1 and PA_HN002, respectively, and overexpressed them in the PA_HN002 strain. If these enzymes were involved in reducing or inhibiting biofilm formation and the mutations were critical for their activity, biofilm formation in PA_HN002 should be greater when it overexpresses its endogenous enzyme compared to the homolog from the PAO1 strain. As shown in **Figure 5B**, overexpression of RbdA, FimX, NbdA, PipA and RmcA from both strains did not display significant differences on the biofilm biomass of PA_HN002, which suggested that mutations in these enzymes were not associated with the hyper-biofilm production in PA_HN002. Interestingly, overexpression of PA3258 from PA_HN002 showed significantly lower biofilm biomass than the PA3258 enzyme from PAO1, indicating the mutations presented in PA3258 of PA_HN002 indeed led to the inhibition of biofilm formation rather than the promotion of biofilm formation as we expected. Since mutations of I112V and A166S were located in the GGDEF domain, it was possible that PA3258 exhibited DGC activity and these two positions I112 and A166 were important for the enzymatic activity.

**Figure 6:**
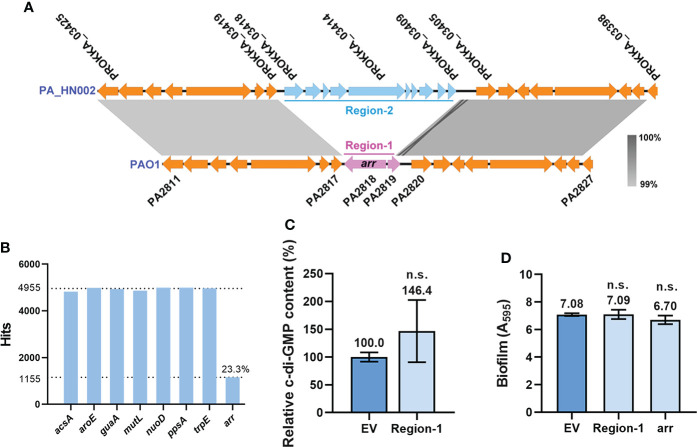
Loss of Arr did not affect intracellular c-di-GMP content and biofilm formation. **(A)** Replacement of the *arr* gene-containing region (Region-1) by a fragment containing 10 coding sequences (Region-2) was detected in the PA_HN002 genome. The light, gray-shaded regions showed more than 99% sequence identity. **(B)** Abundance of the *arr* gene in 4,955 *P. aeruginosa* genomes. A set of house-keeping genes *acsA*, *aroE*, *guaA*, *mutL*, *nuoD*, *ppsA* and *trpE* used for MLST analysis were selected as controls to show their distributions in almost 100% genomes. **(C)** Relative intracellular c-di-GMP content measured by LC-MS in the PA_HN002 strain complemented with the fragment of Region-1 from PAO1. **(D)** Biofilm formation of the PA_HN002 strain and PA_HN002 with the complementation of the Region-1 and overexpression of the *arr* gene. n.s., not significant based on Student’s *t*-test. EV, empty vector.

In addition to the substitutions of amino acids, it was noted that the entire PDE gene *arr* (*PA2818*) was absent in the PA_HN002 genome (**Figure 5A**). This gene together with its upstream sequence (Region-1: 289 bp of the *PA2819* gene and 67 bp of the intergenic region between *arr* and *PA2819*) were replaced by a large 6,879-bp sequence (Region-2) containing 10 genes in PA_HN002 (**Figure 6A**). *arr* was reported only in a very limited number (5 of 20) of *P. aeruginosa* strains and supposed not to be a component of the *P. aeruginosa* core genome (Kulesekara et al., 2006). We also searched Arr orthologs based on its amino acids sequence within 4,955 genomes from different geographic regions and isolation sources in the Pseudomonas Genome Database using DIAMOND BLASTP (Buchfink et al., 2015; Winsor et al., 2016). Consistently, only 1155 (23.3%) hits of Arr were detected in these genomes (**Figure 6B**), indicating the rare distribution of Arr in the clinically and environmentally relevant strains. We next sought to figure out whether missing of Arr in PA_HN002 led to its high intracellular c-di-GMP content and strong biofilm formation. We cloned the whole Region-1 (**Figure 6A**) from the PAO1 genome but found that introduction of this sequence into PA_HN002 to complement Arr expression did not affect the intracellular c-di-GMP content as well as the biofilm biomass (**Figures 6C, D**). Further overexpression of Arr with a constitutive promoter P*lac* did not alter the biofilm biomass of PA_HN002 as well (**Figure 6D**). These results indicated that higher intracellular c-di-GMP content and biofilm formation found in PA_HN002 than PAO1 was not owing to the absence of Arr and the occurrence of identified mutations in the c-di-GMP metabolizing enzymes.

### Differentially expressed c-di-GMP metabolizing enzymes in PA_HN002 compared to PAO1

**Figure 7:**
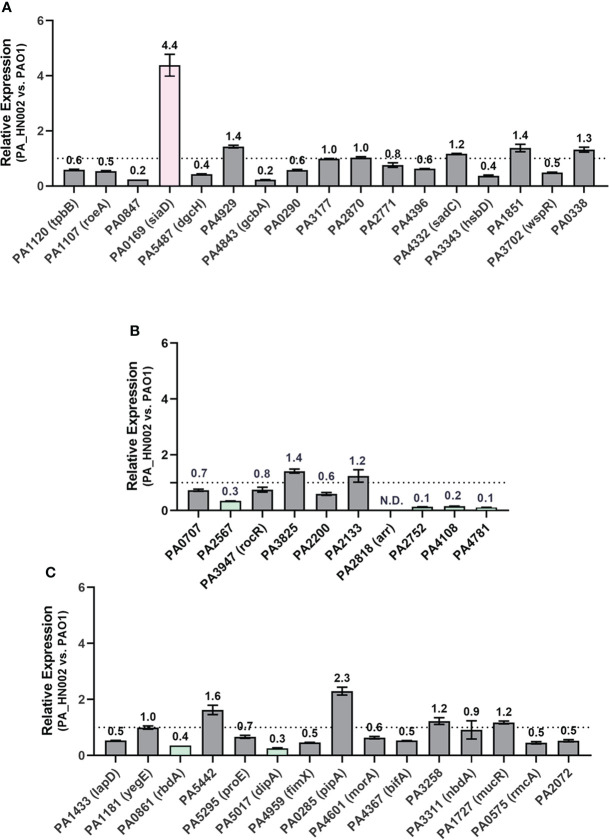
Relative expression of the genes encoding c-di-GMP metabolizing enzymes in PA_HN002 and PAO1. **(A)** Enzymes only contain the GGDEF domain. **(B)** Enzymes contain either EAL or HY-GYP domain. **(C)** Enzymes contain both the GGDEF and EAL domains. The DGC-encoding gene *siaD* in PA_HN002 was highlighted in pink while the genes encoding PDE enzymes with more than 2-fold changes such as PA2567, PA4108, PA4781, PA0861 (*rbdA*), PA5017 (*dipA*) and PA0285 were highlighted in green. N.D., Not detected.

Besides the mutations present in the c-di-GMP hydrolysis genes, an increased level of c-di-GMP biosynthesis and a reduced level of c-di-GMP hydrolysis owing to the changed expression levels of DGC and PDE genes represent additional important factors causing accumulation of intracellular c-di-GMP. To evaluate the expression level of enzymes involved in c-di-GMP metabolism, relative expression of all the c-di-GMP metabolizing genes between PAO1 and PA_HN002 was measured by RT-qPCR. Genes in PA_HN002 with more than 2-fold transcriptional changes relative to their homologs in PAO1, i.e. > 2-fold upregulation of DGC genes and downregulation of PDE genes, were selected for further discussion. For the GGDEF domain-containing DGCs, only the PA0169 (SiaD) was expressed 4.4-fold higher in PA_HN002 than that in PAO1 (**Figure 7A**). Overexpression of SiaD to increase intracellular c-di-GMP content and enhance biofilm formation was demonstrated recently (Zhou et al., 2021). For the EAL or HD-GYP domain-containing PDEs, four enzymes PA2567, PA2752, PA4108 and PA4781 in PA_HN002 besides Arr were expressed at lower levels which were 30%, 10%, 20% and 10% of that in PAO1 (**Figure 7B**). PA2567, PA4108 and PA4781 were confirmed enzymes with PDE activity while PA2752 did not exhibit PDE activity but was implicated with biofilm formation (Ryan et al., 2009; Kimura et al., 2017). For enzymes containing both GGDEF and EAL domains, PA0861 (RbdA) and PA5017 (DipA) in PA_HN002 were expressed at more than 2-fold lower than that in PAO1 (**Figure 7C**). Both enzymes were demonstrated to exhibit PDE activity (An et al., 2010; Roy Ankita et al., 2012). These results showed a dramatically different expression profile of c-di-GMP metabolizing enzymes between PA_HN002 and PAO1, which might be the major cause of the elevated c-di-GMP content and the strong biofilm formation in PA_HN002.

### Mobile genetic elements and unique COGs in PA_HN002

We next sought to investigate the factors altering the expression pattern of c-di-GMP metabolizing enzymes. *P. aeruginosa* genome is highly plastic and complex. It frequently incorporates exogenous sequences by horizontal gene transfers into its genome. These MGEs including genomic islands (GIs), plasmids, integrative and conjugative elements (ICEs), integrative and mobilizable elements (IMEs), insertion sequences (ISs), prophages *etc.* comprise the accessory genome of *P. aeruginosa* (Shen et al., 2006; Silby et al., 2011; Ozer et al., 2014; Subedi et al., 2018). These elements were considered as a major group of contributors to *P. aeruginosa* genome evolution by introducing new catabolic pathways, encoding novel regulators, or disrupting endogenous gene functions and regulatory circuits, conferring the pathogen specific phenotypes to adapt to diverse environmental conditions through shifting the gene expression profile.

To facilitate the understanding of specific regulatory mechanisms of c-di-GMP metabolism in PA_HN002, we analyzed MGEs presented in its genome. Firstly, twenty GIs were identified in the PA_HN002 genome and some genes in the GIs were assigned to functional groups related to gene regulation and signal sensing (**Table S4**). There was an IME element found in the PA_HN002 genome which contained 8 coding genes therein. In addition to an integrase and a relaxase, others were hypothetic proteins. Sequence alignment suggested a DNA-binding protein, a RES domain-containing protein, a sce7725 family protein and a sce7726 family protein therein (**Figure S2**). In total five complete ISs, namely ISPa22, ISPa6, ISPa1, ISPa57 and ISPa32, were found in the genome of PA_HN002 (**Table S5**). Notably, multiple incomplete copies of IS222 were found at the end of several contigs (data not shown). PHASTER analysis revealed four potential prophages with 47.8, 36.2, 32.3 and 7.7-kb in length (**Table S6**). Next, genes with unique COG annotations in the PA_HN002 genome compared to PAO1 were summarized, showing that 54 COGs were identified exclusively in PA_HN002 and they were basically from the MGEs (**Table 1**). Together, presence of the abundant MGEs and unique COGs might account for the changed expression profile of c-di-GMP metabolizing enzymes and strong biofilm formation in PA_HN002.

**Table 1:** COG identified in *P. aeruginosa* PA_HN002 but not in PAO1.

No.	COG ID	Annotation
1	COG1872	Uncharacterized conserved protein YggU, UPF0235/DUF167 family
2	COG3094	Uncharacterized membrane protein SirB2
3	COG3369	Uncharacterized conserved protein, contains Zn-finger domain of CDGSH type
4	COG3423	Predicted transcriptional regulator, lambda repressor-like DNA-binding domain
5	COG2842	Bacteriophage DNA transposition protein, AAA+ family ATPase
6	COG4396	Mu-like prophage host-nuclease inhibitor protein Gam
7	COG4382	Mu-like prophage protein gp16
8	COG5566	Transcriptional regulator, middle operon regulator (Mor) family
9	COG5410	Uncharacterized conserved protein
10	COG4383	Mu-like prophage protein gp29
11	COG2369	Uncharacterized protein, contains phage Mu head morphogenesis gpF-like domain
12	COG4388	Mu-like prophage I protein
13	COG4397	Mu-like prophage major head subunit gpT
14	COG5449	Uncharacterized conserved protein, DUF2163 domain
15	COG2425	Uncharacterized conserved protein, contains a von Willebrand factor type A (vWA) domain
16	COG0287	Prephenate dehydrogenase
17	COG2515	1-aminocyclopropane-1-carboxylate deaminase/D-cysteine desulfhydrase, PLP-dependent ACC family
18	COG4570	Holliday junction resolvase RusA (prophage-encoded endonuclease)
19	COG5377	Phage-related protein, predicted endonuclease
20	COG0582	Integrase/recombinase, includes phage integrase
21	COG3311	DNA-binding transcriptional regulator AlpA
22	COG2995	Intermembrane transporter PqiABC subunit PqiA
23	COG4628	Uncharacterized conserved protein, DUF2132 family
24	COG5460	Uncharacterized conserved protein, DUF2164 family
25	COG3328	Transposase (or an inactivated derivative)
26	COG2323	Uncharacterized membrane protein YcaP, DUF421 family
27	COG2916	DNA-binding protein H-NS
28	COG2732	Barstar, RNAse (barnase) inhibitor
29	COG0270	DNA-cytosine methylase
30	COG5589	Uncharacterized conserved protein, DUF2390 domain
31	COG3805	Aromatic ring-cleaving dioxygenase
32	COG4103	Tellurite resistance protein TerB
33	COG2173	D-alanyl-D-alanine dipeptidase
34	COG1545	Uncharacterized OB-fold protein, contains Zn-ribbon domain
35	COG2068	CTP:molybdopterin cytidylyltransferase MocA
36	COG3575	Uncharacterized conserved protein
37	COG3637	Opacity protein LomR and related surface antigens
38	COG1783	Phage terminase large subunit
39	COG5635	Predicted NTPase, NACHT family domain
40	COG3621	Patatin-like phospholipase/acyl hydrolase, includes sporulation protein CotR
41	COG3410	Uncharacterized conserved protein, DUF2075 family
42	COG4983	Primase-polymerase (Primpol) domain protein
43	COG4823	Abortive infection bacteriophage resistance protein
44	COG1479	DNAse/DNA nickase specific for phosphorothioated or glycosylated phage DNA, GmrSD/DndB/SspE family, contains DUF262 and HNH nuclease domains
45	COG2357	ppGpp synthetase catalytic domain (RelA/SpoT-type nucleotidyltranferase)
46	COG5654	Uncharacterized protein, contains RES domain
47	COG3723	Recombinational DNA repair protein RecT
48	COG3567	Uncharacterized conserved protein, DUF1073 domain
49	COG3566	Uncharacterized conserved protein, DUF2213 domain
50	COG4834	Uncharacterized conserved protein, DUF2184 domain
51	COG1993	PII-like signaling protein
52	COG5662	Transmembrane transcriptional regulator RsiW (anti-sigma-W factor)
53	COG4387	Mu-like prophage protein gp36
54	COG5005	Mu-like prophage protein gpG

## Discussion

*P. aeruginosa* is a highly motile opportunistic pathogen. Its motility is a key determinant for its colonization within the host, which promotes the establishment of numerous acute infections at different human tissues. Loss of motility and inducing the pathogen into a sessile-biofilm lifestyle reflect an adaptative process under human immune pressures or anti-infective interventions (Balloy et al., 2007). The structured biofilm is extremely resistant to antimicrobial-based treatments and eradication of the biofilm pathogen is almost impossible. Unfortunately, genetic determinants that drive the formation of biofilms in clinically significant strains remain largely unknown, which impedes the precise control and prevention of chronic infections in hospitals. PA_HN002 isolated in this study is a representative adapted strain of chronic urinary tract infection, which was phylogenetically close to the strains isolated from different countries such as China, Canada and USA. This strain provided us an important model to investigate how *P. aeruginosa* shifted its lifestyle to persist in the host. Some previous studies have reported that loss of the flagellar structural proteins or the stator complexes in the flagellar motor is an adaptive strategy conferring *P. aeruginosa* resistance to host phagocytosis by neutrophils and macrophages (Amiel et al., 2010; Lovewell et al., 2011). Here, we further demonstrated in PA_HN002 that the increased level of the second messenger c-di-GMP played a critical role in the transition from its motile lifestyle to the sessile biofilm lifestyle. Instead of the complete abolishment of swimming and swarming owing to the loss of functions of flagellar biosynthetic genes, the motile lifestyle in PA_HN002 could be reversed when its intracellular concentration of c-di-GMP was decreased.

High-throughput sequencing technique has facilitated the in-depth characterization of bacterial genetic determinants that contribute to physiological traits or biological processes with interests in the past decades. With the high-throughput genome sequencing technique, we identified a list of mutations in the c-di-GMP metabolizing enzymes in PA_HN002. The potential effects of these mutations on biofilm formation were examined and, however, we found that none of them was associated with the enhanced biofilm formation in PA_HN002. Intracellular c-di-GMP dynamics has been extensively investigated in the past decade and numerous environmental cues or cellular regulatory pathways that modulate intracellular c-di-GMP content have been revealed. For example, Proteins encoded by *siaABCD* regulated c-di-GMP production and biofilm formation in response to the toxic surfactant sodium dodecylsulfate (SDS) in *P. aeruginosa* (Colley et al., 2016; Chen et al., 2020a). We also found recently that the two-component regulatory system FleS/FleR controlled biofilm formation and type VI secretion system by modulating the intracellular c-di-GMP content (Zhou et al., 2021; Zhou et al., 2022). These achievements greatly advanced our understanding in the adaptive mechanisms of *P. aeruginosa* and highlighted the importance of the signaling molecule c-di-GMP during this adaptative process by controlling the transition of lifestyles between motility and sessility in *P. aeruginosa*.

Quantification of the c-di-GMP metabolizing gene expression in PA_HN002 showed lots of them were differentially expressed compared to their homologs in PAO1. Most of these genes with changed expression level were previously confirmed to play roles in regulating biofilm formation. Noticeably, SiaD was substantially overexpressed in PA_HN002. A comprehensive analysis of overexpressing DGC-encoding genes in PAO1 revealed that SiaD and another enzyme SadC were two dominant DGCs to produce remarkably more c-di-GMP than other DGCs (Bhasme et al., 2020). These results together indicated a plausible connection between the overexpression of SiaD and the high intracellular c-di-GMP content in PA_HN002. In addition to Arr, putative PDEs such as PA2567, PA2752, PA4108 and PA4781 were expressed at lower levels than their homologs in PAO1. PA2567 is an azithromycin responsive EAL domain-containing PDE which was repressed in the presence of azithromycin and deletion of PA2567 has been reported to reduce the quorum sensing activity and virulence of the *P. aeruginosa* strain (Kimura et al., 2017). PA4108 and PA4781 are two PDEs that contain a HD-GYP domain rather than the EAL domain. The role of these two PDEs in c-di-GMP hydrolysis was confirmed because mutations of PA4108 and PA4781 led to an increase in the level of c-di-GMP in *P. aeruginosa* (Ryan et al., 2009). Strains with PA4108 and PA4781 mutations showed different biofilm architectures but, interestingly, mutation of PA4108 led to a reduced biofilm biomass (Ryan et al., 2009). PA2572 encodes a non-canonical variant YN-GYP which was reported to display undetectable activity in c-di-GMP degradation but was associated with biofilm formation in *P. aeruginosa* (Ryan et al., 2009). Two more enzymes PA0861 (RbdA) and PA5017 (DipA) containing both the GGDEF and EAL domains but only exhibiting PDE activity were found differentially expressed with more than 2-fold lower compared to their homologs in PAO1 (An et al., 2010; Roy Ankita et al., 2012). Techniques to precisely control the expression of these c-di-GMP metabolizing enzymes and efficiently screen their regulators are highly desired for the in-depth understanding of the special regulation of c-di-GMP metabolism in PA_HN002 and other clinical strains.

*P. aeruginosa* possesses a complicated gene regulatory network to exquisitely control its virulence as well as its survival in response to the diverse environmental conditions. This is heavily dependent on its large genome and the abundant transcriptional factors encoded from it. It was estimated that there are about 600 transcriptional factors encoded from a *P. aeruginosa* genome with many of them are associated with the dynamics of intracellular c-di-GMP content (Stover et al., 2000). Moreover, a *P. aeruginosa* genome is generally composed of a large percentage of accessory genome which includes a large number of MGEs acquired by horizontal gene transfer from different sources (Ozer et al., 2014). These elements and genes therein are known to impact the native expression of host genes as well as the host virulence and antibiotic resistance. In PA_HN002, a set of MGEs and genes with unique COG annotations were identified in its genome. In the future, targeted molecular dissections to unveil the mechanisms on the regulation of c-di-GMP metabolizing enzymes and evolutionary analysis on the transmission of MGEs may provide more clinically relevant insights into the pathoadaptation of *P. aeruginosa* causing chronic infections and help to identify new targets for precise anti-pseudomonal treatments.

Arr is a membrane-associated PDE which was initially found to be essential for aminoglycoside-dependent induction of biofilm formation and reported to promote biofilm formation during the DNA replication stress (Hoffman et al., 2005; Gotoh et al., 2008). These were contradictory to the general concept that PDEs function to reduce c-di-GMP content and therefore prevent biofilm formation (Ha et al., 2015). We initially speculated that loss of Arr in PA_HN002 was a contributor to its high intracellular c-di-GMP content and the hyper-biofilm. However, expression of *arr* in the PA_HN002 strain did not influence its c-di-GMP content. This result suggested that Arr might not be involved in c-di-GMP metabolism despite it contains an EAL domain. Moreover, inconsistent with the previous reports that Arr promoted biofilm formation, we did not observe increased biofilm formation in PA_HN002 with the expression of Arr. This discrepancy indicated that Arr could be a stress-responsive regulator since our examinations were performed under non-stressed conditions.

## Data availability statement

The datasets presented in this study can be found in online repositories. The names of the repository/repositories and accession number(s) can be found below: https://www.ncbi.nlm.nih.gov/genbank/, JAMXFG000000000.

## Author contributions

ZX designed the study; SL, SC, HC, TL, MH, LLiao performed experiments; LLi isolated the clinical strain; SL, SC drafted the manuscript; LLi, L-HZ, ZX revised the manuscript. All authors contributed to the article and approved the submitted version.

## Funding

This work was supported by Guangzhou Basic and Applied Basic Research Foundation (No. 202201010613), National Natural Science Foundation of China (No. 32100020) and Guangdong Basic and Applied Basic Research Foundation (No. 2022A1515010194).

## Conflict of interest

The authors declare that the research was conducted in the absence of any commercial or financial relationships that could be construed as a potential conflict of interest.

## Publisher’s note

All claims expressed in this article are solely those of the authors and do not necessarily represent those of their affiliated organizations, or those of the publisher, the editors and the reviewers. Any product that may be evaluated in this article, or claim that may be made by its manufacturer, is not guaranteed or endorsed by the publisher.
