# Supplementary material for: Genome characterization of a uropathogenic Pseudomonas aeruginosa isolate PA_HN002 with cyclic di-GMP-dependent hyper-biofilm production
Source: Front Cell Infect Microbiol. 2022 Aug 2;12:956445. doi: 10.3389/fcimb.2022.956445 (PMC9394441; doi:10.3389/fcimb.2022.956445)
Supplement: Supplementary file 1 [file DataSheet_1.pdf]

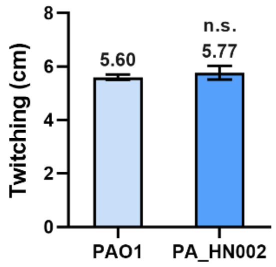

Figure S1. Twitching motility measured in *P. aeruginosa* PAO1 and PA_HN002. n.s.: not significant based on Student's *t*-test.

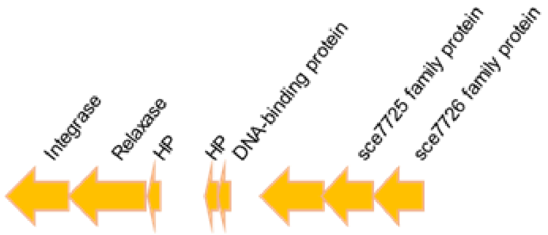

Figure S2. Structure of the IME identified in the PA_HN002 genome.

Table S1. Primers used in this study

Name	Sequence	Description
pBBR-Reg1-F	gtcgacggtatcgataagcttcgaaaaacagttgctcgcgc	for amplification of region-1
pBBR-Reg1-R	cgctctagaactagtggatccggctgctcgtcctggtctc	
pBBR-Arr-F	gtcgacggtatcgataagcttacggacatgaccctgatccc	for amplification of *arr*
pBBR-Arr-R	cgctctagaactagtggatccgactctttgcgcttccgcc	
pBBR-FimX-F	tcgacggtatcgataagcttatggccatcgaaaagaaaaccatcc	for amplification of *fimX*
pBBR-FimX-R	gctctagaactagtggatcctcattcgtctcccgaggagaag	
pBBR-NbdA-F	tcgacggtatcgataagcttatgccttttctccccggga	for amplification of *nbdA*
pBBR-NbdA-R	gctctagaactagtggatcctcaggcctggttcaggct	
pBBR-PipA-F	tcgacggtatcgataagcttatgagcccccgcctgagt	for amplification of *pipA*
pBBR-PipA-R	gctctagaactagtggatcctcagtcttccggcagcgcc	
pBBR-PA3258-F	tcgacggtatcgataagcttgtgaaatacggggccggtt	for amplification of *PA3258*
pBBR-PA3258-R	gctctagaactagtggatcctcaggcggcctcgatcag	
pBBR-RbdA-F	tcgacggtatcgataagcttatgaggcagaaccggactct	for amplification of *rbdA*
pBBR-RbdA-R	gctctagaactagtggatccctaccggaggttctgtcccag	
pBBR-RmcA-F	tcgacggtatcgataagcttatgcagcgtctgcaggcc	for amplification of *rmcA*
pBBR-RmcA-R	gctctagaactagtggatccttataccggcgcttctcggg	
pBBR-veri-F	tacgcaaaccgcctctcccc	for verification of the constructs
pBBR-veri-R	gctgcgcaactgttgggaag	
PA1120-F	ccagttggcatcgggaaa	For qPCR

PA1120-R	cgtgcaggatcggttgtt	
PA1107-F	cttcaacctgatccacatcct	For qPCR
PA1107-R	atgggcgtacatgatttcct	
PA0847-F	cgcttcgacatcaccaagaa	For qPCR
PA0847-R	aggtagccgaccaggaaa	
PA0169-F	tcttcaagcaggtcaacgatac	For qPCR
PA0169-R	gccgcagaggtcgtattc	
PA5487-F	agcgcgacttcgaggaata	For qPCR
PA5487-R	tgtccagcacgtcggtatag	
PA4929-F	cacgacgaactgctggaata	For qPCR
PA4929-R	ggtcagcacgtaccagaaatag	
PA4843-F	cctgtacaaccacacccatac	For qPCR
PA4843-R	tcgttgacctgcttgaagtg	
PA0290-F	gaggacatcaccgacaagaag	For qPCR
PA0290-R	tcggcactgtcgaagaaatag	
PA3177-F	ccaagcattcggtgagctatc	For qPCR
PA3177-R	agttgccgttgttcgtcttc	
PA2870-F	ggcggaactcatgctcaa	For qPCR
PA2870-R	tggctgtcgttgatgttctt	
PA2771-F	gcccttcatccgcttctac	For qPCR
PA2771-R	gattgagctggcgtcctt	
PA4396-F	tgcagcagctcgaacaa	For qPCR
PA4396-R	gacgatgtaggtgtagtggttg	
PA4332-F	ctggctgacctatttcctctac	For qPCR
PA4332-R	ttggaacacgccgaaca	
PA3343-F	tcagtttcgccgcctatttc	For qPCR
PA3343-R	accactcgaaacccaggta	
PA1851-F	catcctcgcggtgttctt	For qPCR
PA1851-R	gttggtacgcaggttctctt	
PA3702-F	cttcgacgaatacctggagatg	For qPCR
PA3702-R	gaaggtgtcgttgtagctctt	
PA0338-F	tcatccatcccgaggactat	For qPCR
PA0338-R	atcgctgatccacaggtaatc	
PA1433-F	cggcaaccttacccatctg	For qPCR
PA1433-R	gatagaccgcttcgatgaacaa	
PA1181-F	cggcctgagttccttcaatta	For qPCR
PA1181-R	gttgatggagtcgacgatgg	
PA0861-F	cgtttctccctggacgattt	For qPCR
PA0861-R	ctgccatcgatcttcaggtaat	
PA5442-F	ggaaatctccgtgctgatga	For qPCR
PA5442-R	gcggaagaactggaaggtatt	
PA5295-F	gtccttcgtccacctcaatag	For qPCR
PA5295-R	acttccaggttgaggttgtg	
PA5017-F	caagaaccacgagccttact	For qPCR
PA5017-R	gtcttcgtagatgccgatgtag	

PA4959-F	cgtttcggcgattcgatctt	For qPCR
PA4959-R	ggtggttctcgactttcttcag	
PA0285-F	gcgttcctgagcgatcattt	For qPCR
PA0285-R	ttgcggcgaagaagaacag	
PA4601-F	gaactgagcctgctgatgaa	For qPCR
PA4601-R	tcatctcggcctggtagaa	
PA4367-F	cccgccctatagcgaatacta	For qPCR
PA4367-R	gagaatgccggagatgaagatg	
PA3258-F	ccagcgtatcgaggaagattt	For qPCR
PA3258-R	aagcaaggtgagcggatt	
PA3311-F	tggctcccgctatctatact	For qPCR
PA3311-R	aggccttgccaatccatag	
PA1727-F	gtgatcctcgagcagttgtc	For qPCR
PA1727-R	ggcaggcgtttgagataca	
PA0575-F	ccactactgcatgagcgaaag	For qPCR
PA0575-R	ccaaatcgcgaatccagactac	
PA2072-F	gcagttctccaccacctattc	For qPCR
PA2072-R	ccgaggttgttcttggtgaa	
PA0707-F	acgtcgacaatgaggaactg	For qPCR
PA0707-R	cctggaaaggctccgaatag	
PA2567-F	ggacgaggtattcgaggaaatc	For qPCR
PA2567-R	tgatggtcgaggatggaaatg	
PA3947-F	aatttccctggtggtggaag	For qPCR
PA3947-R	ccggagcaatagtcgagaaag	
PA3825-F	aggtggactgcctgaagat	For qPCR
PA3825-R	cgacgatgctgtcgagaatatg	
PA2200-F	ctgaagatctacgtggacgattt	For qPCR
PA2200-R	gatggactgagtgaagaccttg	
PA2133-F	gcatcgcgatagacgactt	For qPCR
PA2133-R	gtataccggcaatcgatcttga	
PA2818-F	gtaacctctgtcgccattct	For qPCR
PA2818-R	atggtcatccgggtcaatc	
PA2752-F	ctcgcctatgaggagctaaag	For qPCR
PA2752-R	gatgatttgctggttggtctg	
PA4108-F	cattccggtaacccagctt	For qPCR
PA4108-R	gcaggaagccacctcttt	
PA4781-F	cgaatacctggaactcgaagtg	For qPCR
PA4781-R	gctcgatacgcggaagatg	

Table S4. Genomic islands (GIs) identified in PA_HN002

GI No.	Gene locus tag range	Contig No.
GI-1	PROKKA_05272 - PROKKA_05286	NODE_8
GI-2	PROKKA_00594 - PROKKA_00608	NODE_10

GI-3	PROKKA_00708 - PROKKA_00723	NODE_10
	PROKKA_00894 - PROKKA_00896	NODE_10
GI-4	PROKKA_03001 - PROKKA_03011	NODE_27
	PROKKA_00543 - PROKKA_00549	NODE_1
GI-5	PROKKA_00363 - PROKKA_00372	NODE_1
GI-6	PROKKA_00133 - PROKKA_00145	NODE_1
GI-7	PROKKA_05862 - PROKKA_05868	NODE_9
GI-8	PROKKA_05689 - PROKKA_05742	NODE_9
GI-9	PROKKA_01677	NODE_14
GI-10	PROKKA_02838 - PROKKA_02841	NODE_22
GI-11	PROKKA_03014 - PROKKA_03015	NODE_28
GI-12	PROKKA_00957 - PROKKA_00964	NODE_11
GI-13	PROKKA_03492 - PROKKA_03502	NODE_3
GI-14	PROKKA_03452 - PROKKA_03460	NODE_3
GI-15	PROKKA_04331 - PROKKA_04340	NODE_5
GI-16	PROKKA_02570 - PROKKA_02575	NODE_2
GI-17	PROKKA_02262 - PROKKA_02278	NODE_2
GI-18	PROKKA_01801 - PROKKA_01804	NODE_16
	PROKKA_02194 - PROKKA_02200	NODE_2
GI-19	PROKKA_01916 - PROKKA_01918	NODE_16
	PROKKA_04024	NODE_48
GI-20	PROKKA_04466	NODE_54
	PROKKA_04879	NODE_65

Table S5. List of insertion sequences (ISs) identified in PA_HN002

IS No.	IS name	Family	Identity (%)	Start	End	Length (bp)	Contig No.
1	ISPa22	IS1182	99.88	152178	153841	1664	NODE_1
2	ISPa6	ISNCY	96.86	399755	400230	476	NODE_1
3	ISPa1	ISNCY	98.75	402555	403587	1033	NODE_1
4	ISPa57	IS3	96.96	5209	6384	1176	NODE_12
5	ISPa32	IS3	99.92	2994	4229	1236	NODE_27

Table S6. List of prophages identified in PA_HN002

ID	Score	Total protein	Reference phage	Start position/coordinates on the contig	End position/coordinates on the contig	Contig No.
1	140	55	phi297	426921	474739	NODE_3
2	70	46	JBD93	280242	316528	NODE_5
3	150	39	YMC11/02/R656	16554	48910	NODE_8
4	96	11	Pf1	62263	69965	NODE_10
